# Robotic assisted surgery reduces ergonomic risk during minimally invasive colorectal resection: the VOLCANO randomised controlled trial

**DOI:** 10.1007/s00423-024-03322-y

**Published:** 2024-04-27

**Authors:** Frances Dixon, Parveen Vitish-Sharma, Achal Khanna, Barrie D. Keeler, on behalf of the VOLCANO Trial Group, Adnan Qureshi, Anjana Singh, Anil Hemandas, Richard O’Hara, Lynn Wren, Santos Oshiotse, Susan George

**Affiliations:** 1 Department of Surgery, Milton Keynes University Hospital NHS Foundation Trust, Milton Keynes, Eaglestone, MK6 5LD UK; 2 University of Buckingham, Buckingham, MK18 1EG UK

**Keywords:** Ergonomics, Colorectal, Robotic Surgery, Minimally Invasive Surgery

## Abstract

**Purpose:**

Minimally invasive surgery benefits patients but poor operating ergonomics causes musculoskeletal injuries in surgeons. This randomised controlled trial aims to assess whether robotic-assisted surgery with the open-console Versius® system can reduce surgeons’ ergonomic risks during major colorectal resections.

**Methods:**

Prospectively registered at ClinicalTrials.gov (NCT05262296) in March 2022. Adult patients requiring a minimally invasive colorectal resection were potentially eligible. Photographs taken at 2-min intervals were analysed using the objective Rapid Entire Body Assessment (REBA) posture analysis scale to calculate intraoperative surgeon ergonomic risk. Secondary outcomes included team communication (Oxford NOTECHS II), surgeon cognitive strain (modified NASA-TLX scale), and clinical outcomes.

**Results:**

Sixty patients were randomised in a 2:1 ratio (40 robot, 20 laparoscopic). Mean age was 65yrs and 34 (57%) were male. Body Mass Index did not differ between the 2 groups (overall mean 29.0 ± 5) and there were equal proportions of left and right-colonic resections.

REBA was significantly lower in the robotic arm (median robot REBA score 3 vs lap REBA 5 [p < 0.001]), equating to an injury risk category drop from “medium” to “low risk”. There were no significant differences in team communication, operative duration, or patient outcomes. Surgeon cognitive strain was lower in robotic cases (mean robot 32.4 ± 10.3 vs lap 45.6 ± 14.3 [p < 0.001]).

**Conclusions:**

This trial demonstrates that robotic surgery with an open-console system reduces ergonomic risk scores and cognitive strain during colorectal resections, with no apparent detriment to team communication. This may therefore be a safe & feasible solution to the increasing problem of work-related musculoskeletal injuries in surgeons.

## Introduction

Minimally invasive surgery is now recognised as the gold standard surgical modality for treating colorectal cancer, given improved outcomes when compared to open surgery [1]. However, although the benefits to patients are well described, laparoscopic surgery has been shown to be harmful to the surgeons who perform it. Up to 90% of surgeons report experiencing pain attributable to performing laparoscopic surgery, and this not only has short-term effects but may lead to high numbers seeking early retirement [2, 3]. Finding solutions to improve surgeons’ intraoperative ergonomics is essential and investigation of robotic surgery as a potential solution is indicated [4].

Robotic surgery is an evolution of laparoscopic surgery intended to expand the ability to perform minimally invasive operations, and is increasingly utilised in colorectal surgery [5]. Robotic consoles have been designed with ergonomic principles in mind which may allow for lower physical demand on surgeons, an assertion reflected in the anecdotal experience of a number of surgeons [6]. This is supported by a meta-analysis on the ergonomics of robotic surgery, though this was limited by being largely based on self-reported surveys or small-scale studies in simulated environments [7]. Improved surgeon ergonomics may support the increased adoption of robotics, and offset the increased costs associated with this modality but there is a lack of randomised data assessing the ergonomics of robotic surgery [8].

Robotic systems can be “closed” or “open” console design. A closed console is one where the surgeon places their head into the viewfinder whereas an open console has a flat screen and the surgeon’s head is free (most often used in conjunction with 3D glasses). All prior research on ergonomics in robotic surgery has been conducted using closed console systems which generally have favourable ergonomics but have been reported to necessitate nonergonomic flexion of the neck [9]. Seated surgery can easily be adapted for optimal ergonomics, but the pedal position on existing robotic systems may lead to leg extension and core instability [10]. This trial aims to assess the ergonomics of an open console pedal-less system – the Versius® system from CMR Surgical (Cambridge, UK).

This is the first trial to assess whether robotic-assisted surgery using an open console system can reduce surgeons’ ergonomic risk scores during major colorectal resectional surgery, and uses a validated outcome measure and randomised design to objectively assess robotic surgical ergonomics. Secondary endpoints include intraoperative team communication, surgeon cognitive strain and patient clinical outcomes.

## Methods

### Trial design

This was a single centre, randomised, unblinded trial comparing robotic-assisted with laparoscopic assisted major colorectal resectional surgery. Ethical approval was obtained (IRAS 305564), and the trial was prospectively registered at ClinicalTrials.gov (NCT05262296) and undertaken in accordance with the Declaration of Helsinki and Consolidated Standards of Reporting Trials (CONSORT) guidelines [11]. Major colorectal resection was defined as any operation requiring removal of part or all of the colon or rectum, with an anastomosis or formation of a stoma.

### Participants

Any adult patient requiring a minimally invasive colorectal resection who was discussed in the local colorectal cancer or inflammatory bowel disease multidisciplinary meetings and had no previously assigned lead clinician was deemed eligible for inclusion in the trial. Patients requiring emergency surgery, multi-visceral resections, pregnant patients, and those unable to consent were not eligible. Clinical review was undertaken on all potentially eligible patients prior to recruitment to ensure suitability for inclusion. Neither patients nor surgeons received any compensation for participation, and all participants provided written consent.

### Intervention

This trial compared laparoscopic major colorectal (resectional) surgery with robotic. All robotic surgery was conducted using the Versius® platform. Exact surgical technique was at the discretion of the operating surgeon. Both modalities are currently offered as standard at the host institution and surgeons participating in either arm of the trial were required to have previously performed at least 20 cases using the relevant modality.

### Outcomes

**Fig. 1:**
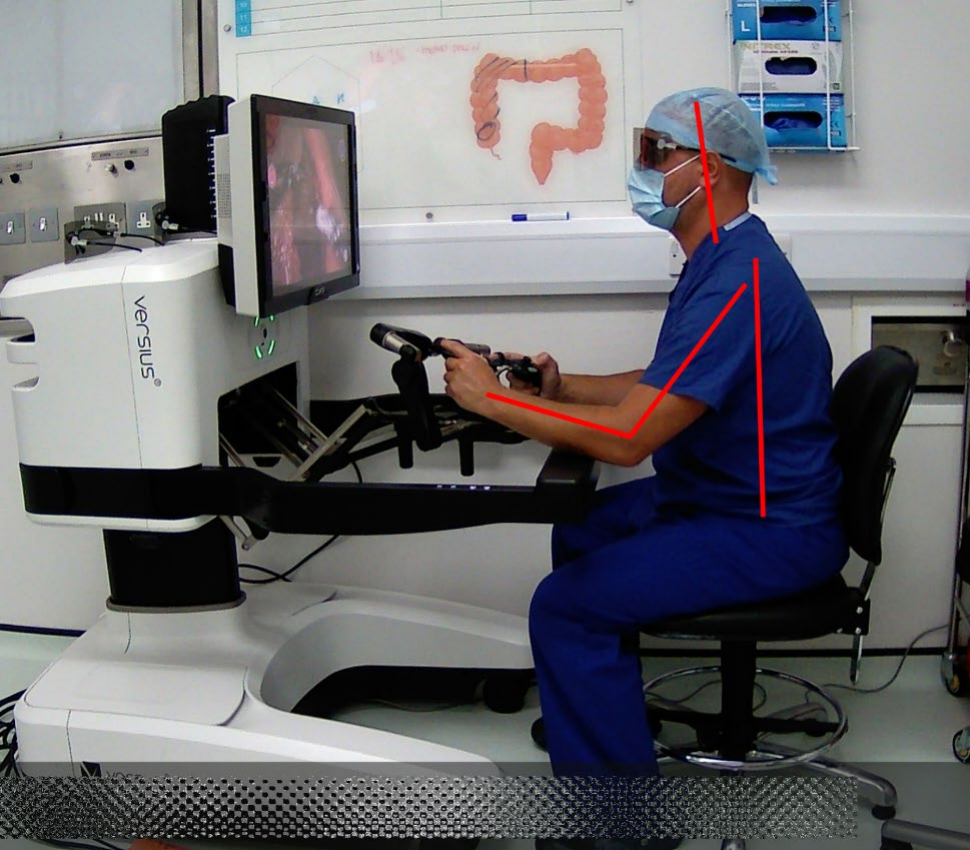
Example Photograph for REBA (Rapid Entire Body Assessment) Analysis

The primary endpoint was operating surgeon ergonomic risk, as measured by the Rapid Entire Body Assessment (REBA) tool and calculated from intraoperative photographs of the surgeon. REBA is an objective postural analysis system that attributes scores to relative positions of different body parts, based on risk of injury. It has been widely used and validated across a number of industries but was specifically intended to be sensitive to the “unpredictable working postures found in healthcare” [12, 13]. The raw ergonomic risk score generated (from 1 to 15) corresponds to injury risk categories: from “negligible risk” to “very high risk”, which give an indication of how quickly that posture should be changed. For example, a “very high risk” posture should be changed immediately to reduce the chance of lasting injury (see Appendix 1 for all categories and action levels). A step-by-step guide to calculating REBA is available from www.ergo-plus.com. REBA was calculated from intraoperative photographs taken of the operating surgeon at 2-min intervals throughout each operation and analysed using the validated open-source FIJI software (ImageJ v1.54f) [14]. Figure 1 shows a representative photograph with superimposed lines demonstrating some of the analysed joint angles. Two cameras were placed to ensure an unobstructed view was obtained, with the optimal photograph analysed after surgery in each instance.

The prespecified secondary endpoints included surgeon cognitive strain, intraoperative team communication, and patient clinical outcomes. The NASA Task Load Index (NASA-TLX) is a widely used and validated cognitive load assessment tool and was modified to also include 3 questions from the Surgery Task Load Index (SURG-TLX), making a total of 9 questions, each scored on a scale from 1–10 [15, 16]. Subdomains assessed in the questionnaire are mental, physical, and temporal demand, task complexity, situational stress, effort required, frustration, environmental distraction, and self-assessment of performance. Raw scores rather than weighted were used for each subdomain, giving a total score out of 90 [17]. The surgeon was asked to complete the questionnaire immediately after the end of each operation by a member of the research team, using an electronic form to minimise difficulties in data interpretation.

Intraoperative team communication was assessed using the Oxford NOTECHS II, which is a validated method for evaluating the non-technical skills of the entire operating team, sub-divided into surgical, anaesthetic, and nursing teams [18]. Each sub-team is rated from 1–8 in 4 domains: leadership and management, teamwork and cooperation, problem-solving and decision-making, and situation awareness. Scores were calculated from live in-theatre observation by a researcher with a clinical background who has been trained in the assessment of surgical non-technical skills. Patient outcomes included intraoperative factors (operative time, estimated blood loss), postoperative factors (recovery measures, length of stay, pain score on day 1, 2, 3, and 28) and histological outcomes, with all data collected from medical records or directly from patients. Trial completion point was at 28 days postoperatively, after a follow up telephone call from the research team.

### Sample size

Prior studies of surgeon posture have shown an average REBA of 6.9 for laparoscopic surgery [10]. In order to demonstrate a reduction in REBA from 6.9 to 5.7 with 80% power at a 5% significance level (2-sided), a total sample size of 60 patients was targeted, which also accounted for anticipated drop-outs (up to 20%). This projected reduction was calculated based on local pilot data in non-clinical settings, in conjunction with prior studies of REBA in robotic-assisted surgery using other robotic platforms [19–21]. As per the distribution-based approach for calculating minimum clinically importance differences, as the anticipated degree of change in REBA score is greater than 0.5 standard deviations this would indicate a clinically significant difference between the two operative techniques [22].

### Randomisation

Patients were randomised using variable block randomisation in a 2:1 ratio (2 robotic:1 laparoscopic) using software from ALEA (https://www.aleaclinical.eu/) which allowed for concealment of allocation from the research team. This ratio was selected given the already well-established nature of laparoscopic surgery and also as a continuation of the existing allocation ratios in the host institution, based on surgeon skill-mix. Stratification was performed using body mass index (BMI; ≤ 30 kg/m^2^ and > 30 kg/m^2^) and side of resection. Right-sided resections were defined as between caecum and splenic flexure, and left-sided from descending colon to anal canal.

### Blinding

Blinding of patient participants was not undertaken as there were no subjective patient outcomes assessed in this trial. It was not possible to blind surgeon or theatre team participants, or members of the research team who analysed photographs, to performed modality.

### Statistical methods

All results were analysed on an intention-to-treat basis, with all tests 2-tailed and at a 5% level of significance. Normality tests were carried out on data and subsequent analyses determined by data distribution. Descriptive statistics were reported and chi-square tests used, along with Mann–Whitney U for non-parametric data and t tests for parametric data. A weighted Cohen’s kappa was calculated to assess inter- and intra-rater reliability of photograph analysis. All analyses were performed using SPSS Version 29.0 (IBM Corp, Armonk, NY, USA).

## Results

**Fig. 2:**
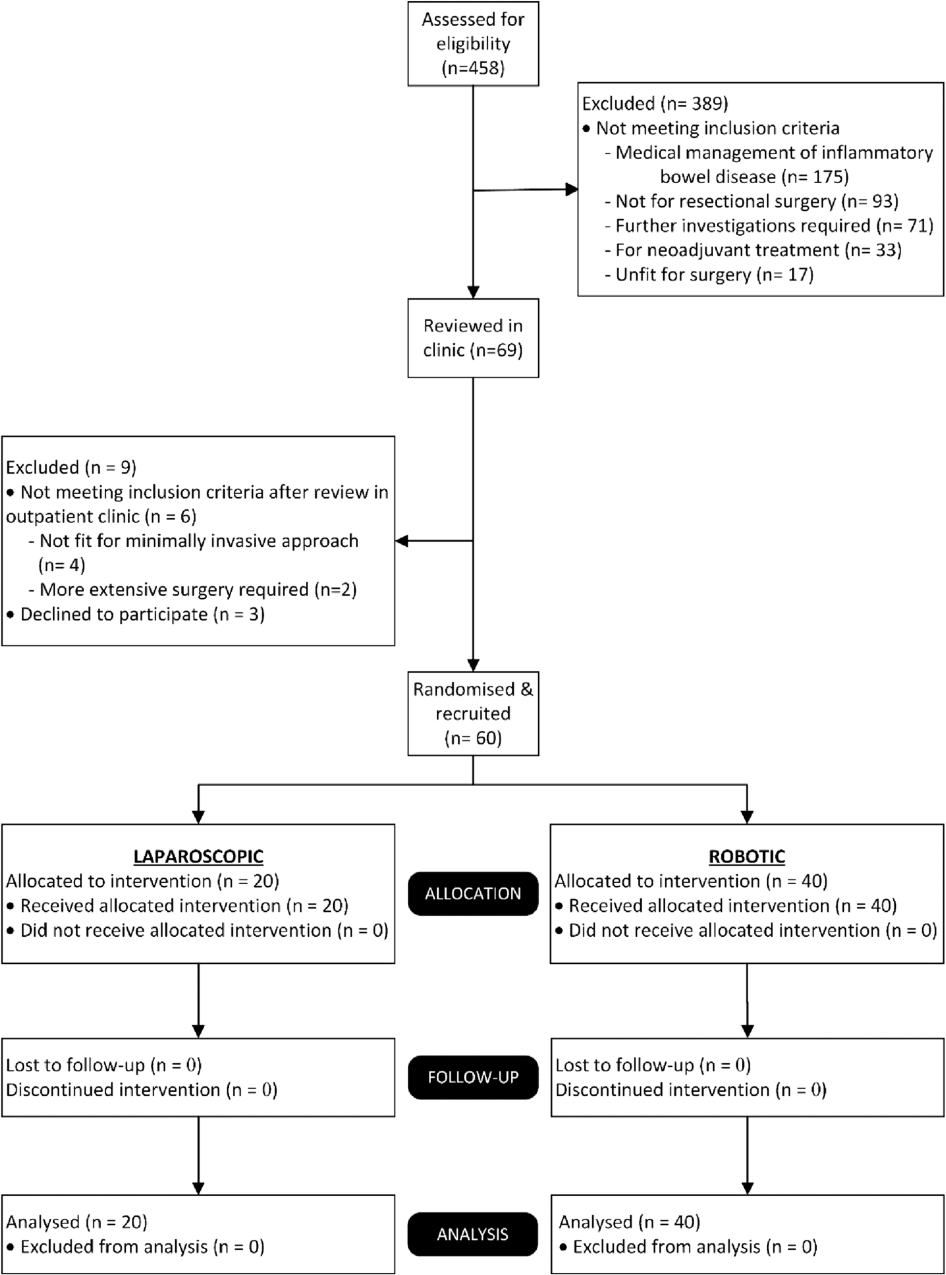
Patient Flow (CONSORT) Chart

Between August 2022 and March 2023, 458 patients were screened for eligibility and 69 were deemed eligible for the trial (see Fig. 2 for CONSORT flow diagram). Of the 60 patients randomised for inclusion in the trial, all underwent surgery via their allocated modality, none were lost to follow up and all were included in the final analysis. The trial was closed following completion of the final patient follow up, 10 months after the trial opened. A total of six surgeons performed all cases in the trial; three of whom performed cases in both arms of the trial.

As demonstrated in Table 1, the baseline demographics, patient characteristics, and operative indications were similar between the two groups, as were intraoperative and postoperative outcomes (Table 2). Each group only had one complication rated higher than a Clavien-Dindo grade II. In the robot arm, one patient required a radiologically-guided drain insertion into a perineal wound collection (Grade IIIa) and one laparoscopic patient was admitted to the critical care unit following a reoperation for small bowel perforation secondary to ileus (Grade IVa). The most common Grade I and II complications across both groups were wound infection (n = 7 [robot = 4, lap = 3]), postoperative pyrexia (n = 3 [robot = 3, lap = 0]), and chest infection (n = 3 [robot = 2, lap = 1]).

**Table 1:** Participant Baseline Demographics

	**Robot (n = 40)**	**Laparoscopic (n = 20)**
Age, mean ± SD (range)	65.8 ± 11.2 (40–84)	64.2 ± 14.5 (37–84)
Sex ratio (Male:Female)	23:17 (58% male)	12:8 (60% male)
Ethnicity, n (%) - White - Black or Black British - Asian or Asian British	39 (97.5%) 1 (2.5%) 0 (0%)	18 (90%) 1 (5%) 1 (5%)
BMI (kg/m^2^), mean ± SD	28.7 ± 4.2	29.6 ± 5.1
Smoking status, n (%)		
- Never - Former - Current	31 (77.5%) 6 (15%) 3 (7.5%)	15 (75%) 4 (20%) 1 (5%)
Indication, n (%)		
- Cancer - Advanced polyp - Diverticular disease - Lymphoma	35 (87.5%) 4 (10%) 1 (2.5%) 0 (0%)	17 (85%) 2 (10%) 0 (0%) 1 (5%)
Charlson comorbidity score, median (IQR)	4 (2)	4 (3)
ASA score		
- 1 - 2 - 3	2 (5%) 29 (72.5%) 9 (22.5%)	0 (0%) 13 (65%) 7 (35%)
Prior abdominal surgery, n (%)		
- Yes - No	16 (40%) 24 (60%)	9 (45%) 11 (55%)

SD = standard deviation, IQR = interquartile range, ASA = American Society of Anesthesiologists

**Table 2:** Postoperative and Clinical Outcomes

	**Robot** **(n = 40)**	**Laparoscopic (n = 20)**	**p value**
**Intraoperative:**			
Left-sided, n (%) Of which,	24 (60%)	11 (55%)	0.83
- Anterior resection - Abdominoperineal resection - Left hemicolectomy - Hartmann’s	14 (35%) 8 (20%) 1 (2.5%) 1 (2.5%)	8 (40%) 1 (5%) 1 (5%) 1 (5%)	
Right-sided (right hemicolectomy), n (%)	16 (40%)	9 (45%)	
Conversion from planned modality, n (%)	0 (0%)	0 (0%)	NA
Total op time (minutes), mean ± SD	296.7 ± 98.7	263.9 ± 91.0	0.21
Estimated blood loss (millilitres), median (IQR)	100 (0)	100 (0)	0.86
**Postoperative:**			
Pain score, median (IQR)			
- Day 1 post-op - Day 2 - Day 3 - Day 28	0 (4) 0 (4) 0 (2) 1 (2)	0 (4) 0 (3) 0 (0) 0.5 (2)	0.83 0.79 0.34 0.62
Length of stay (days), median (IQR)	6 (2)	6 (3)	0.79
Recovery indicators, median (IQR):			
- Time to passage of flatus (days) - Sustained resumption of normal diet (days) - Return to baseline mobility (days)	3 (2) 3 (1) 3 (3)	3 (1) 4 (2) 4 (3)	0.64 0.16 0.99
Return to normal preoperative activity levels at 28 days, n (%)	4 (10%)	0 (0%)	0.14
Readmission within 30 days, n (%)	2 (5%)	1 (5%)	NA
**Complications:**			
Complication during primary admission, n (%)	6 (15%)	2 (10%)	0.59
Clavien-Dindo grade, n (%)			
- I - II - IIIa - IIIb - IVa	2 (5%) 4 (10%) 0 0 0	0 1 (5%) 0 0 1 (5%)	
Complication post-discharge (< 30 days), n (%)	7 (17.5%)	6 (30%)	0.27
Clavien-Dindo grade, n (%)			
- I - II - IIIa - IIIb	1 (2.5%) 5 (12.5%) 1 (2.5%) 0	1 (5%) 5 (25%) 0 0	

SD = standard deviation, IQR = interquartile range

The median robotic operative REBA score was 3 (IQR 1), compared to 5 (IQR 0) in the laparoscopic arm (p < 0.001). This equates to a drop in risk category from “Medium” to “Low” risk (Appendix 1). A subset (n = 100) of photographs were re-analysed by the same researcher after a period of 4 weeks, and analysed by a second researcher. Weighted Cohen’s kappa demonstrated that there was almost perfect intra-rater agreement (κ = 0.84 [p < 0.001]) and substantial inter-rater agreement (κ = 0.79 [p < 0.001]) [23, 24].

Overall cognitive strain was also lower in the robotic group (mean modified NASA-TLX score 32.4 ± 10.3 SD vs 45.6 ± 14.3 SD [p < 0.001]). Table 3 shows this significance was also reflected in the scores for the mental demand, physical demand, temporal demand, situational stress, and performance subdomains. The task complexity, effort, frustration, and distraction subdomains did not demonstrate a difference. There was no difference in intraoperative team communication between the two modalities, with mean Oxford NOTECHS II scores of 72.6 ± 3.7 SD in the robotic arm and 71.6 ± 3.9 SD in the laparoscopic arm (p = 0.33). There were no differences shown between surgical, anaesthetic, or nursing sub-team scores. The surgical team’s “situational awareness” subdomain was specifically analysed, given prior reports of the impact of robotic surgery on situational awareness, and this showed no difference (p = 0.76). Although team communication was assessed by a live in-theatre observer, surgeons in all cases reported being “unaware” of being observed and stated that they did not change their behaviour in response to being observed.

**Table 3:** Ergonomic Outcomes

	**Robot** **(n = 40)**	**Laparoscopic (n = 20)**	**p value**
Operative REBA, median (IQR)	3 (1)	5 (0)	< 0.001*
Total modified NASA-TLX, mean ± SD	32.4 ± 10.3	45.6 ± 14.3	< 0.001*
- Mental demand subdomain, mean ± SD - Physical demand subdomain, mean ± SD - Temporal demand subdomain, mean ± SD - Task complexity subdomain, mean ± SD - Situational stress subdomain, mean ± SD - Performance subdomain, mean ± SD - Effort subdomain, mean ± SD - Frustration subdomain, mean ± SD - Distraction subdomain, mean ± SD	4.0 ± 1.8 2.1 ± 1.4 2.4 ± 1.7 6.2 ± 1.8 2.6 ± 1.6 2.6 ± 1.2 4.5 ± 1.9 4.2 ± 2.5 4.0 ± 2.6	6.3 ± 2.4 7.0 ± 2.6 4.0 ± 2.1 6.3 ± 2.2 4.2 ± 2.0 2.4 ± 1.6 6.1 ± 2.2 5.5 ± 2.3 3.9 ± 2.0	< 0.001* < 0.001* 0.005* 0.66 0.003* 0.013* 0.06 0.82 0.28
Total Oxford NOTECHS II, mean ± SD	72.6 ± 3.7	71.6 ± 3.9	0.33
- Surgeon sub-team, mean ± SD - Anaesthetic sub-team, mean ± SD - Nursing sub-team, mean ± SD	25.5 ± 2.1 22.8 ± 1.8 24.3 ± 2.2	24.9 ± 1.9 22.8 ± 2.0 23.9 ± 2.5	0.26 0.92 0.47

IQR = interquartile range, SD = standard deviation

Thirty-seven robotic patients and eighteen laparoscopic patients were found to have evidence of cancer on postoperative histology. There were no differences in the proportions of T or N stages between groups (p = 0.80 and 0.78, respectively). All mesorectal dissection specimens were histologically grade 3. One (2.7%) robotic patient had a positive margin due to a tumour deposit. Two (11.1%) laparoscopic patients had a positive margin (tumour deposit and close margin in a patient post maximal neoadjuvant therapy). This difference was not significant (p = 0.20). The median lymph node yield was higher in the robotic group (19 (IQR 11) vs 15.5 (IQR 6) [p = 0.03]).

## Discussion

This is the first randomised controlled trial that examines the ergonomics of surgery using the open console Versius® robotic surgery system, and objectively demonstrates that the use of robotic assistance with an open console can significantly lower surgeon ergonomic risk category from “Medium” to “Low”. These results could indicate previously under-recognised benefits of robotic surgery and may justify the increased cost relative to conventional laparoscopic surgery.

All prior research conducted into robotic surgery has used closed console systems as these were the only commercially available models until recently. Van’t Hullenar et al. report that the limited range of console adjustment with these models means that very short or very tall surgeons may struggle to achieve an optimal ergonomic operating position, and the position of the eyepiece requires neck angulation when operating [9]. This was reflected in a review of the ergonomics of robotic surgery which demonstrated that although overall strain was lower with robotic surgery compared to laparoscopic, surgeons’ neck and trapezius muscles were disproportionately affected [7]. A further small-scale study by Dwyer et al. involving 4 robotic surgeons reported an average REBA of 7 with harmful neck flexion in 3/4 of the participating surgeons, which is interestingly higher than the laparoscopic REBA scores demonstrated in this trial [21].

Multiple survey-based studies describe lower self-reported strain in robotic surgeons, but there is a dearth of randomised controlled studies comparing the ergonomics of the two modalities, and a high risk of bias described in existing studies [25]. This trial was randomised to eliminate patient selection bias and stratification factors were chosen to minimise confounding factors, such as high BMI, which has been shown to increase ergonomic strain during laparoscopic surgery [26].

The choice of open console in the design of Versius® has been reported as being specifically intended to improve team communication [6] but may also have benefits for surgeon ergonomics. Feedback was sought from surgeons and height adjustment to facilitate either a seated or standing operating position was included, with further modifications to allow for surgeons at the extremes of height [6]. Other robotic systems such as the Hugo™ system (Medtronic, Minneapolis, MN, USA) also utilise the open console design.

Robotic surgery requires a different operating theatre layout from laparoscopic or open surgery, with the surgeon remote from the bedside, often with their head in a closed console, which may adversely affect team communication and subsequently increase cognitive strain [27, 28]. This is a disputed topic, with trials reporting both higher and lower strain with robotic surgery. The RIVAL Trial of laparoscopic vs robotic inguinal hernia repair demonstrated higher frustration levels with robotic approaches, whilst other studies have shown lower cognitive strain, particularly for novice surgeons [29, 30]. No difference in cognitive strain or intraoperative team communication was found in this trial. It has also been postulated that the physical distance between patient and surgeon may also compromise surgeon situational awareness [27]. This trial demonstrated no difference in either surgical sub-team communication scores (i.e. communication between console and bedside surgeons), or specifically the surgical team’s “situation awareness” domain score.

Although REBA is a well-established, validated, and widely used scale, the implementation of it as a measure of surgeon posture differs widely. Some studies use a “worst posture” approach with a single image taken at the point at which the surgeon’s posture is most unfavourable [20], whereas others take more regular photographs or video but for only a proportion of the operation [21, 31]. We believe that our approach of taking a photograph every 2 min for the entire duration of the operation provides a true picture of the real-world ergonomic risk of an operation, rather than focusing only on brief instances of extremely abnormal posture. This may explain why our overall laparoscopic case scores (REBA 5) were lower than those that have been previously demonstrated in trials of laparoscopic surgery [10]. The approach used in this trial also takes account of the laparoscopic set-up portion of the robotic operations which has previously been reported to be often performed with very poor ergonomics, and therefore avoids giving falsely low scores to the robotic cases [10].

REBA includes risk categories along with corresponding “action levels”, which describe what action needs to be taken in order to avoid injury and indicate how long a particular posture can be safely maintained[13]. In this trial, a drop from “medium risk” (“Action *is* necessary”) to “low risk” (*“*Action *may be* necessary”) was demonstrated.

None of the patient outcomes assessed in this trial showed any difference between the two modalities. Although the study was not powered specifically for these outcomes, this demonstrates that robotic surgery does not cause any detriment to patients. There seems to be a mixed picture in the literature when it comes to the clinical outcomes of robotic vs laparoscopic surgery for colorectal pathology. Several meta-analyses disagree on whether robotic surgery decreases complication rates and length of stay when compared to laparoscopic [32–34]. Operating time hass generally been shown to be longer in robotic cases which impacts the overall cost per operation [8, 29]. In this series there was no difference in operative times between groups, although average procedure duration was long in both groups. In summary, this trial demonstrates that robotic surgery does not lead to worse outcomes for patients, and it may be significantly less physically strenuous for surgeons.

### Limitations

This study was undertaken in a single centre which may limit its external validity, although the pragmatic design is intended to counteract this. Although there were sixty patient participants in the trial, all operations were performed by six surgeons. Three surgeons performed cases using both modalities, and the remaining three operated in one arm of the trial only (2 laparoscopic, 1 robotic). Although this spread gives some balance between arms it is possible that if one surgeon had particularly poor ergonomics this may have unduly influenced the results. Similarly, although a minimum of 20 prior cases was specified for each participating surgeon, actual prior experience (particularly robotic) varied between surgeons. The exact number of cases needed to overcome the initial learning curve is still debated but more experienced robotic surgeons have been shown to have improved economy of movement which may also affect ergonomic risk scores [35, 36]. The main outcome measured used in this trial (REBA) was objective but the NASA-TLX cognitive strain measure is subjective and therefore potentially liable to differences in individual surgeons’ ratings. On a similar note, the Hawthorne effect cannot completely be eliminated despite surgeons reporting that they did not change their behaviour from usual.

## Conclusion

This trial demonstrates that robotic surgery with an open console system can reduce surgeons’ ergonomic risk scores and overall risk category when performing major colorectal resectional surgery. This may also reduce cognitive strain without any apparent detriment to intraoperative team communication or patient clinical outcomes. Robotic surgery may be a safe and feasible solution to the increasing problem of work-related musculoskeletal injuries in surgeons.

## Data Availability

No datasets were generated or analysed during the current study.

## Appendix 1

REBA Risk Categories Table 4.

**Table 4:** After Hignett 2000 [13]

REBA Score	Risk Level / Category	Action Required (including further assessment)
1	Negligible	None necessary
2–3	Low	Change of posture may be necessary
4–7	Medium	Change is necessary
8–10	High	Change is necessary soon
11–15	Very High	Change is necessary NOW

## Declarations

### Trial registration

Prospectively registered at ClinicalTrials.gov (NCT05262296) in March 2022.

No material from other sources has been reproduced in this manuscript.
